# Expression characterization and cross-species complementation uncover the functional conservation of *YABBY* genes for leaf abaxial polarity and carpel polarity establishment in *Saccharum spontaneum*

**DOI:** 10.1186/s12870-022-03501-3

**Published:** 2022-03-17

**Authors:** Zeyuan She, Xiaoyi Huang, Mohammad Aslam, Lulu Wang, Maokai Yan, Rongjuan Qin, Yingzhi Chen, Yuan Qin, Xiaoping Niu

**Affiliations:** 1 Guangxi Key Laboratory of Sugarcane Biology, State Key Laboratory for Conservation and Utilization of Subtropical Agro-Bioresources, College of Agriculture, Guangxi University, Nanning, 530004 China; 2 College of Life Science, Fujian Provincial Key Laboratory of Haixia Applied Plant Systems Biology, Fujian Agriculture and Forestry University, Fuzhou, 350002 China; 3 Fishery Multiplication Management Station of Lijiang River Water Supply Hub Project, Guilin, 541001 China

**Keywords:** Sugarcane, *YABBY* genes, Expression analysis, Polarity establishment

## Abstract

**Background:**

Cell polarity establishment and maintenance is indispensable for plant growth and development. In plants, the YABBY transcription factor family has a distinct role in leaf asymmetric polarity establishment and lateral organ initiation. However, for the important sugar crop *Saccharum*, little information on *YABBY* genes is available.

**Results:**

In this study, a total of 20 sequences for 7 *SsYABBY* genes were identified in the sugarcane genome, designated as *SsYABBY1-7* based on their chromosome locations, and characterized by phylogenetic analysis. We provided a high-resolution map of *SsYABBYs’* global expression dynamics during vegetative and reproductive organ morphogenesis and revealed that *SsYABBY3/4/5* are predominately expressed at the seedling stage of stem and leaf basal zone; *SsYABBY2/5/7* are highly expressed in ovules. Besides, cross-species overexpression and/or complementation verified the conserved function of *SsYABBY2* in establishing leaf adaxial-abaxial polarity and ovules development. We found that the *SsYABBY2* could successfully rescue the leaves curling, carpel dehiscence, and ovule abortion defects in *Arabidopsis crc* mutant.

**Conclusions:**

Collectively, our study demonstrates that *SsYABBY* genes retained a conserved function in establishing and preserving leaf adaxial-abaxial polarity and lateral organ development during evolution.

**Supplementary Information:**

The online version contains supplementary material available at 10.1186/s12870-022-03501-3.

## Background

Establishing cell polarity, asymmetric division, and determining cell fates are essential phases in organ formation and development [1–3]. Polarity establishment and maintenance is a result of polarity formation initiated by a polarizing signal [2]. For example, the HD-ZIP III REVOLUTA (*REV*) and KANADI (*KAN1*) regulate leaf abaxial-adaxial polarity in *Arabidopsis* [4]. Auxin Response Factors (*ARF3* and *ARF4*) and miR166, together with *KANADI* and *YABBY* genes, control the abaxial cell fate identity in *Arabidopsis* [5–7]. Previous studies demonstrated that YABBY transcription factors are essential for polarity establishment and maintenance [8, 9]. YABBY proteins contain a C2C2 domain and a YABBY domain [10–12], and are classified into five different groups (*FIL*/*YAB3*, *YAB2*, *YAB5*, *INO*, and *CRC* subgroups) in several plant species [12].

In *Arabidopsis*, *FIL/YAB3*, *YAB2*, and *YAB5* redundantly regulate lateral organs development [11]. The triple mutant *yab135* (*fil-8 yab3-2 yab5-1*) and quadruple mutant *yab1235* (*fil-8 yab2-1 yab3-2 yab5-1*) lacked apical dominance, and loss of lamina expansion and polarity [13, 14]. In rice, the *FIL* ortholog *TONGARI-BOUSHI* (*TOB1*, *TOB2*, and *TOB3*) also showed conserved functions in flower meristems and lateral organ primordia [15]. *INO* controls the outer ovule integument development in *Arabidopsis*. The *ino-1* mutant exhibits the absence of the outer integument and the typical hoodlike structure characteristic of wild-type ovules, suggesting that *INO* participates in the polar determination of abaxial identity in the ovule [11, 12, 16]. *CRC* is involved in establishing carpels polarity and nectary specification in *Arabidopsis* [11]. The *CRC* ortholog in rice, *DL* (*DROOPING LEAF*) mutation causes carpels completely transformed into stamens [17]. A similar phenotype of carpel morphogenesis was also observed in the ortholog of *CRC* in peas [18, 19]. While in maize, the *CRC* homolog gene *DRL1* (*Drooping Leaf1*) expressed in incipient and emergent leaf primordia functions modulating plant architecture [20–22]. In tomatoes, *SlYABBY2b* regulates fruit size by controlling carpel number during flowering and fruit development [23, 24]. Additionally, *AaYABBY5* promotes artemisinin biosynthesis by increasing the expression of artemisinin biosynthesis genes (*ADS*, *CYP71*, *AV1*, *DBR2* and *ALDH1*) in *Artemisia annua* [25].

Sugarcane is an economically important Poaceae family crop that produces around 80 % of the world's sucrose and has a market worth of approximately $150 billion/year [26]. Sugarcane cultivars are mainly hybrids derived from its progenitor species, *S. officinarum* and *S. spontanuem* [27, 28]. Sexual propagation is based on the normal growth of male and female gametophytes, which could considerably improve sugarcane quality and heterogeneity of generations. Due to reproductive organ degeneration, little progress has been achieved in sugarcane germplasm improvement by sexual propagation. *YABBY* genes have a wide range of roles in shoot apical and floral meristems; however, it is unclear how YABBY proteins operate in reproductive organs and leaf development in sugarcane.

This study performed the genomic analysis of gene phylogeny, gene structure, and expression patterns of *YABBY* genes during sugarcane leaf and ovule development. We have provided comprehensive information on the sugarcane *YABBY* genes and determined the critical role of *SsYABBY2* in leaf and ovule development. Our findings imply that sugarcane *YABBYs* control leaf polarity development and may also participate in ovule development.

## Results

### Identification and characterization of *YABBY* genes in *S. spontaneum*

A total of 27 candidate *YABBY* gene sequences were identified using HMM search (PF04690) in the sugarcane genome. The SMART and Pfam programs were further used to check the accuracy of SsYABBY member sequences, and 7 sequences that lack a complete YABBY domain were removed. Finally, 20 *SsYABBY* genes, including their alleles, were selected for detailed analysis. According to their chromosomal positions, we designated these genes as *SsYABBY1*-*SsYABBY7*. The detailed information of SsYABBY proteins is listed in Table 1, including gene accession number, chromosomal position, protein length, MW, pI, and numbers of exons. The length of putative SsYABBYs ranged from 333 (SsYABBY3-2) to 1467 (SsYABBY7-2) amino acids with the MW ranging from 12476.3 Da to 51860.6 Da, whereas the pI of SsYABBYs ranged from 7.15 (SsYABBY7-5) to 11.08 (SsYABBY6) (Table 1).

**Table 1:** Protein information of *YABBY* genes in *Saccharum spontaneum*

Gene	Allele-Gene	Sequence ID	Chr	Length(aa)	MW(da)	pI	CDS(bp)	Exon	start	end
*Sspon.01G0021070*	*SsYABBY1-1*	*Sspon.01G0021070-1A*	Chr1A	921	32305.6	9.58	307	7	77569893	77574288
*Sspon.01G0036050*	*SsYABBY2-1*	*Sspon.01G0036050-1B*	Chr1B	687	25650.6	8.67	229	5	17011774	17016306
	*SsYABBY2-2*	*Sspon.01G0036050-2C*	Chr1C	621	23090.3	8.36	207	7	12799109	12804404
	*SsYABBY2-3*	*Sspon.01G0036050-3D*	Chr1D	621	23169.5	8.49	207	7	13778473	13783338
*Sspon.01G0045340*	*SsYABBY3-1*	*Sspon.01G0045340-1B*	Chr1B	573	21462.2	8.71	191	6	89407404	89415254
	*SsYABBY3-2*	*Sspon.01G0045340-2C*	Chr1C	507	19350.7	7.95	169	5	86082510	86090043
	*SsYABBY3-3*	*Sspon.01G0045340-3D*	Chr1D	507	19350.7	7.95	169	5	85015352	85022738
*Sspon.02G0023930*	*SsYABBY4-1*	*Sspon.02G0023930-1A*	Chr2A	516	19063.4	9.05	172	6	81577511	81584953
	*SsYABBY4-2*	*Sspon.02G0023930-2C*	Chr2C	516	19113.5	9.05	172	6	85701005	85708820
	*SsYABBY4-3*	*Sspon.02G0023930-3D*	Chr2D	519	19110.4	9.05	173	6	77486705	77494165
*Sspon.02G0031740*	*SsYABBY5-1*	*Sspon.02G0031740-1A*	Chr2A	597	22079.1	8.50	199	6	1.16E+08	1.16E+08
	*SsYABBY5-2*	*Sspon.02G0031740-2B*	Chr2B	594	22083.0	8.82	198	4	1.1E+08	1.1E+08
*Sspon.02G0034850*	*SsYABBY6*	*Sspon.02G0034850-2D*	Chr2D	624	22480.4	11.08	208	3	9344804	9346224
*Sspon.05G0006300*	*SsYABBY7-1*	*Sspon.05G0006300-1P*	Chr4A	756	26638.1	8.03	252	7	21126304	21128864
	*SsYABBY7-2*	*Sspon.05G0006300-2P*	Chr4B	1467	51860.6	7.99	489	9	17854695	17858735
	*SsYABBY7-3*	*Sspon.05G0006300-3P*	Chr4D	1389	50520.7	9.29	463	14	22048663	22064754
	*SsYABBY7-4*	*Sspon.05G0006300-1A*	Chr5A	804	28454.1	7.16	268	7	20233424	20235465
	*SsYABBY7-5*	*Sspon.05G0006300-2B*	Chr5B	810	28694.5	7.15	270	7	13917880	13920056
	*SsYABBY7-6*	*Sspon.05G0006300-3C*	Chr5C	813	28681.4	7.16	271	7	11612288	11614310
	*SsYABBY7-7*	*Sspon.05G0006300-4D*	Chr5D	804	28454.1	7.16	268	7	21351112	21353152

**Fig. 1:**
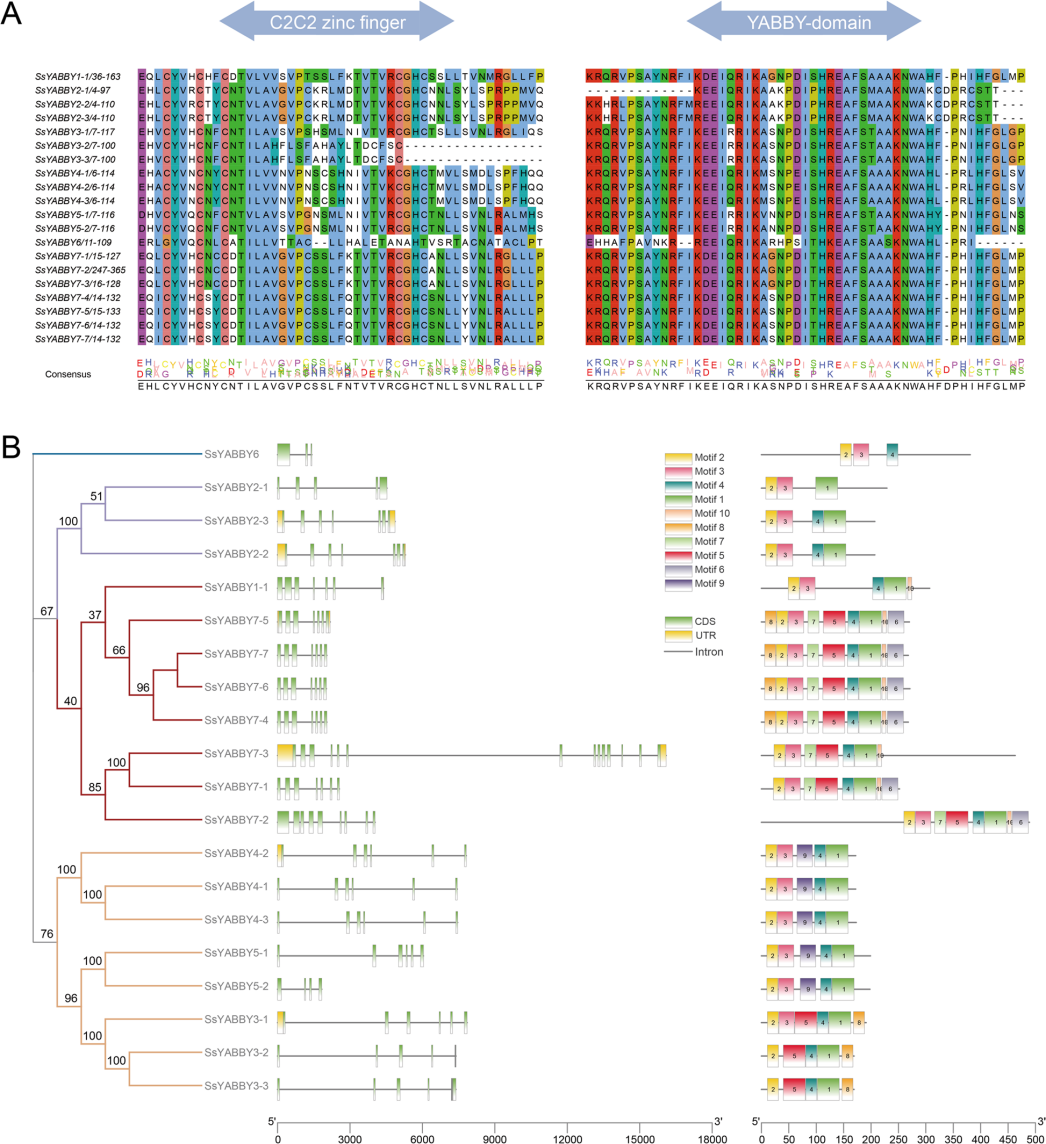
Conserved domains of *YABBY* gene family in *S. spontaneum.* **A** Members of the *YABBY* gene family are characterized by two highly conserved domains: a C2C2 zinc finger domain in the N-terminal and a YABBY domain in the C-terminal. **B** Phylogenetic relationship and gene structure of 7 *SsYABBY* genes. Exons are indicated as green boxes, UTRs in yellow, and black lines linking two exons represent introns

The characteristics of SsYABBY proteins were investigated using 20 SsYABBY protein sequences and aligning them to deduce their domains. Like other YABBY proteins found in plants, all SsYABBYs contain two conserved DNA-binding domains: a C2C2 zinc finger domain and a YABBY domain (Fig. 1A). *SsYABBY3-2* and *SsYABBY3-3* have incomplete C2C2 domains. *SsYABBY6* shows more variability in C2C2 and YABBY domains, indicating its functional diversity (Fig. 1A). A phylogenetic tree of SsYABBYs further demonstrates the conservation of SsYABBYs amino acids and divided them into four subfamilies (*FIL/YAB3*, *YAB2*, *CRC*, and *INO* subfamilies). These subfamilies share a similar exon-intron gene structure. For example, the *YAB2* subfamily possesses six exons with five introns, while *FIL/YAB3* subfamily has seven exons with six introns (Fig. 1B).

### Phylogenetic analysis, gene duplication, and synteny analysis

**Fig. 2:**
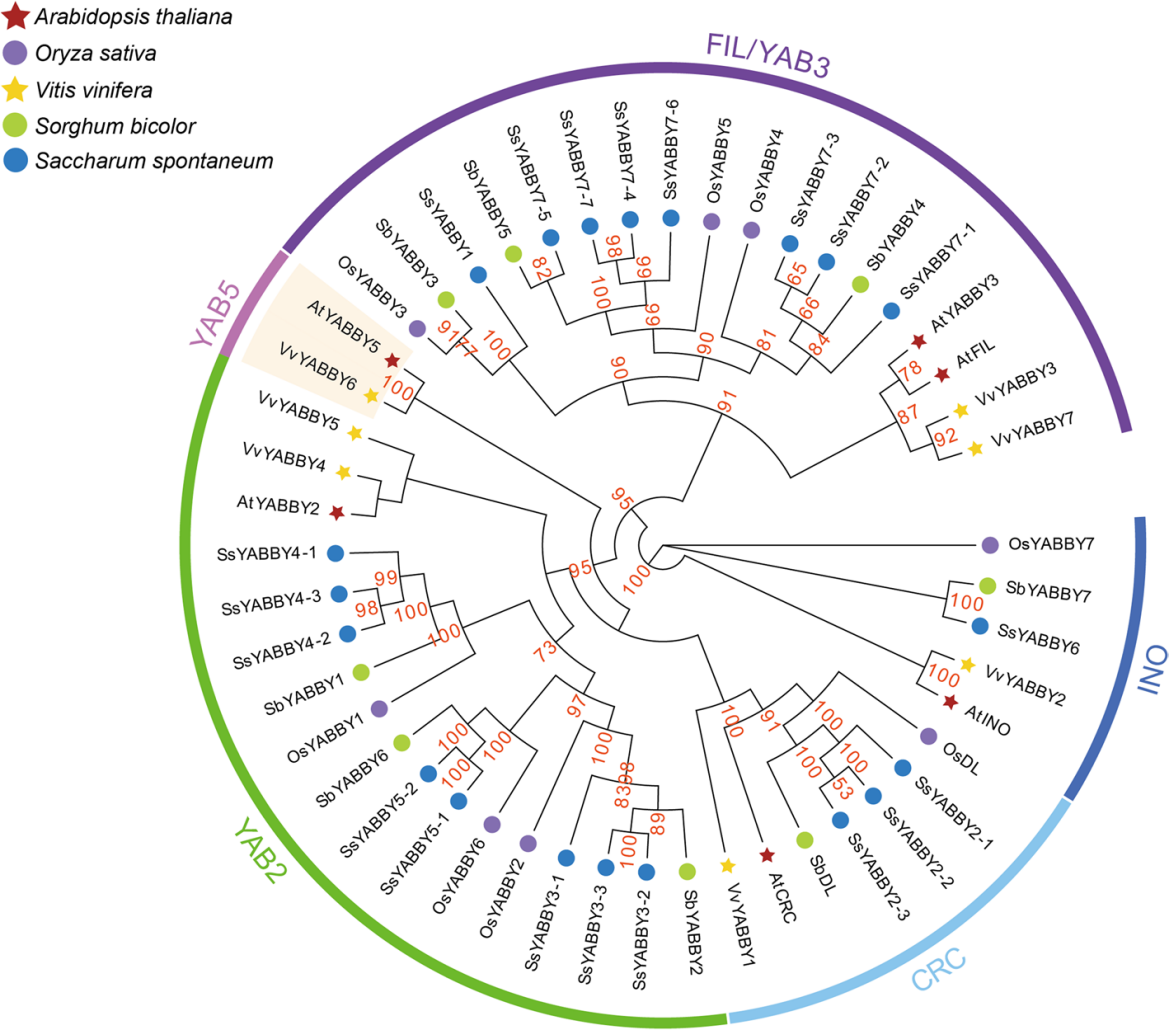
The phylogenetic tree of *YABBY* genes in different species indicates that *YABBY* genes can be clustered into five groups. The ML tree was constructed using MEGA software (version 7.0). The outer circle is marked in dark purple, dark blue, light blue, green, and purple representing the *FIL/YAB3*, *INO*, *CRC*, *YAB2*, and *YAB5* subgroups, respectively. The prefixes *At*, *Os*, *Vv*, *Sb*, and *Ss* represent *A*. *thaliana*, *O*. *sativa*, *V*. *vinifera*, *S*. *bicolor*, and *S. spontaneum* respectively. Bootstrapping was used to test the tree, and only values >60 are displayed

**Fig. 3:**
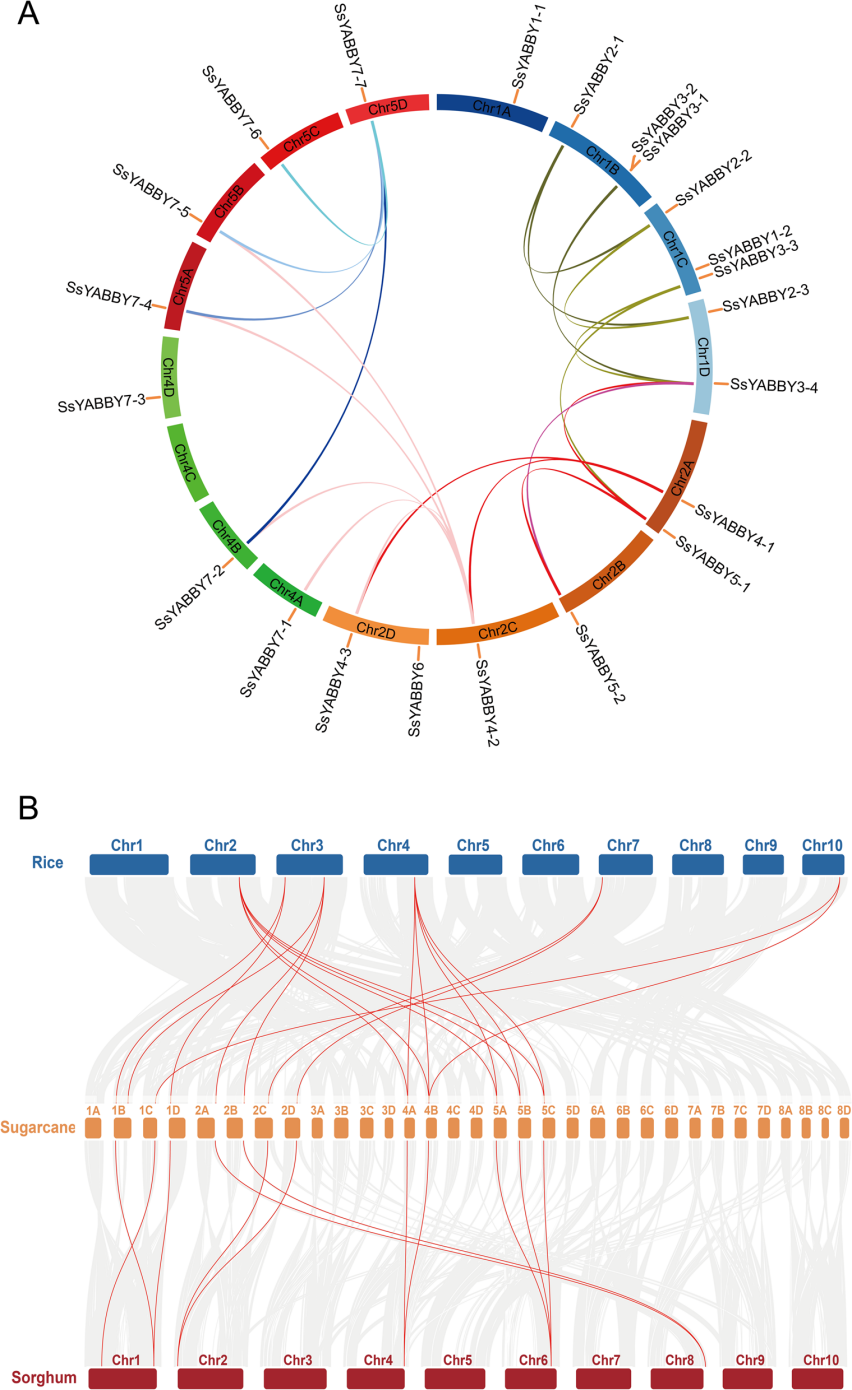
Collinearity analysis for all *SsYABBYs*. **A** SsYABBY genes anchored to corresponding positions on *S*.*spontaneum* chromosomes, as shown in different colors, were analyzed for their duplications. **B** Synteny analysis among the *S*. *spontaneum*, *O. sativa*, and *S. bicolor*

A comprehensive phylogenetic tree was constructed using the maximum-likelihood (ML) method with 20 alleles of *SsYABBYs* and 29 *YABBYs* from monocots and dicots to investigate the evolutionary relationship between sugarcane and other plants. The phylogenetic tree displayed that the *SsYABBY* genes could be classified into four clades: *FIL/YAB3* clade, *YAB2* clade, *YAB5* clade, *CRC* clade, and *INO* clade (Fig. 2). The *SsYABBY* genes, as expectedly, were clustered with the genes of *S*. *bicolor* and *O*. *sativa*, indicating a closer relationship to monocotyledon. The *FIL/YAB3* clade contained the most YABBY members (19), followed by the *YAB2* clade (18), *CRC* clade (7), *INO* clade (5), and *YAB5* clade (2). The *INO* clade had only one gene for each species, and a similar event was also observed in *CRC* clade except for *SsYABBY2* alleles. This result indicated that these *YABBY* members might play similar biological functions. Surprisingly, no *SsYABBY* genes or monocotyledon *YABBY* genes belonged to the *YAB5* clade. In contrast, the *FIL/YAB3* clade and *YAB2* clade contained more *YABBY* genes, showing an obvious gene expansion (Fig. 2). These results indicated that *YABBY* genes gained functional diversity during their species evolution. Also, 20 syntenic gene pairs were identified by MCScanX software, with 13 allele pairs and 7 nonallelic pairs (Fig. 3A; Table S1). We found only one tandem duplication (*SsYABBY3-1*/*SsYABBY3-2*), indicating that segmental duplication is the most common method for the *SsYABBY* gene expansion. The Ka/Ks ratios were calculated to estimate the selection pressure of these homologous gene pairs to better comprehend the evolutionary force of *SsYABBYs*. The results showed that *Ka*/*Ks* ratios of all *SsYABBY* homologous genes were less than 1 (Table S1), indicating that *SsYABBY* genes might experience strong purifying selective pressure during their evolution.

To better understand the evolutionary mechanism of *SsYABBY* genes, the comparative syntenic blocks were constructed between *S. spontaneum* and two monocotyledons *S. bicolor* and *O. sativa*. A total of 19 syntenic orthologous gene pairs were identified between *S. spontaneum* and *O. sativa* (Fig. 3B; Table S2), showing multiple *SsYABBY* genes matched one *OsYABBY* gene. For *S. spontaneum* and its most relative *S. bicolor*, 12 syntenic orthologous gene pairs were found offering two or three *SsYABBYs* syntenic with one *SbYABBY* (Fig. 3B; Table S3).

### Subcellular localization analysis of SsYABBY proteins

**Fig. 4:**
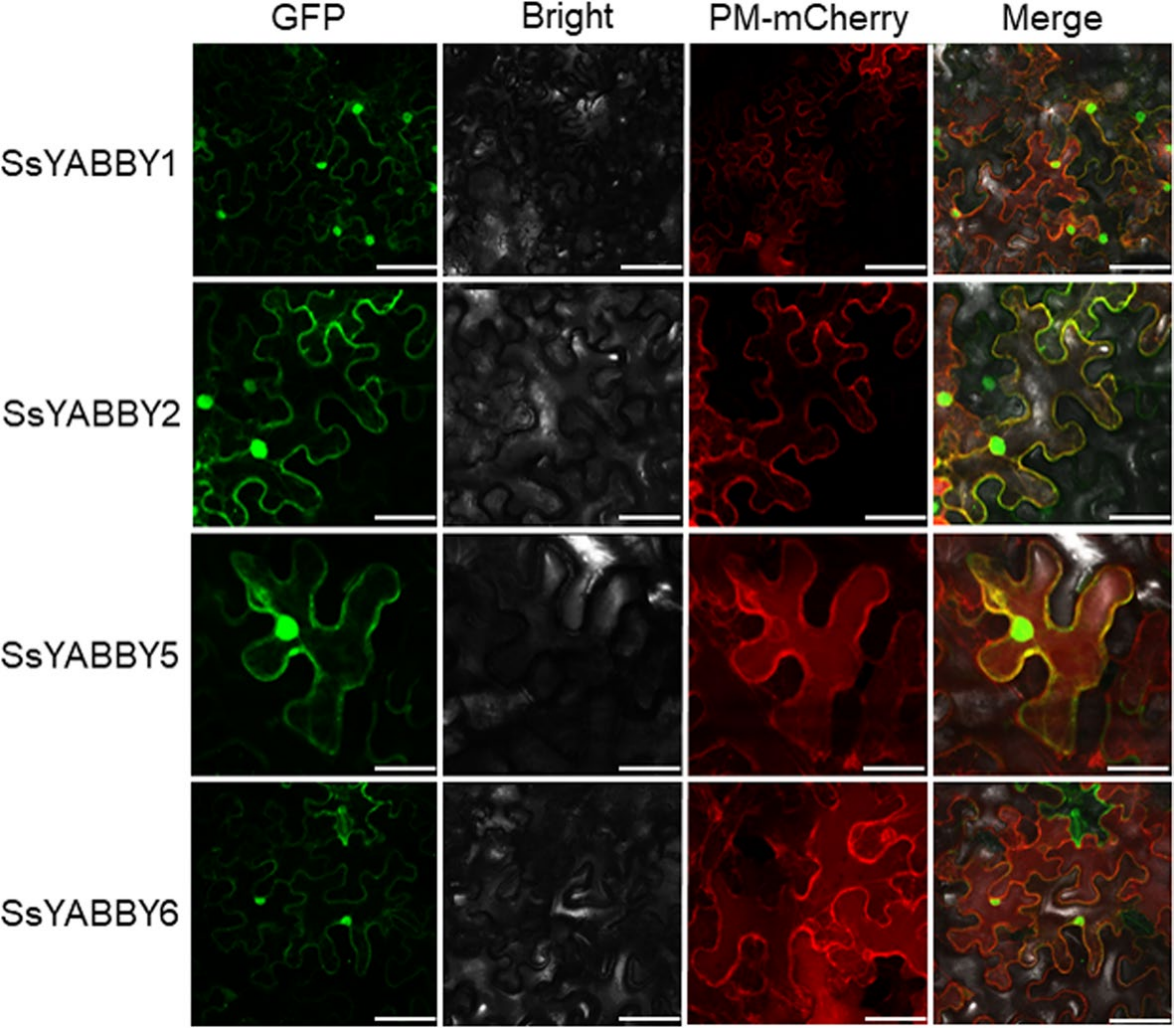
Subcellular localization analysis of SsYABBY1/2/5/6. The subcellular analysis showed that the 35S::*SsYABBY1/2/5/6*-GFP fusion protein was localized in the cell nucleus and membrane. PM-mCherry is a plasma membrane marker. Scale bars: 50 μm

To investigate the molecular characteristics of *SsYABBYs*, four representative *SsYABBY* genes (*SsYABBY1*, *SsYABBY2*, *SsYABBY5*, and *SsYABBY6*) from each subfamily were selected for further subcellular localization analysis based on their phylogenetic relationship. As expected, the GFP signals of *SsYABBY1/2/5/6*-GFP showed that these SsYABBY proteins were nucleus-localized, which is consistent with the previous reports (Fig. 4). Interestingly, the GFP signal of SsYABBY1/2/5 was also detected in the cell membrane, and the fluorescence signal could be well co-localized with that of the membrane marker PM-mCherry (Fig. 4). These results indicated the functional diversity of *SsYABBYs* in the membranes and nucleus.

### Expression profiles of SsYABBYs in different tissues and development stages

**Fig. 5:**
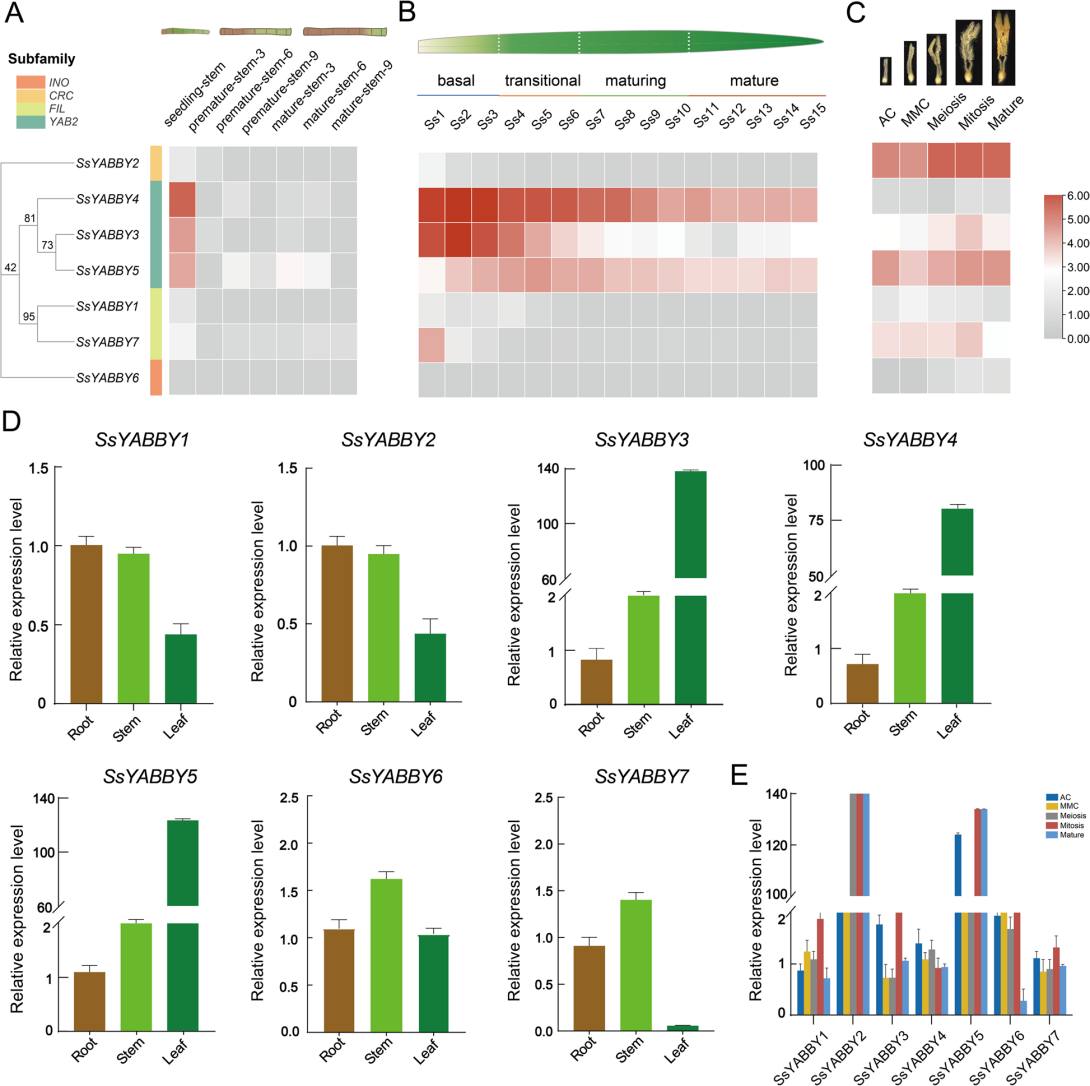
Expression pattern of *YABBY* genes across stems, leaf segments, and different stages of ovule development in *S. spontaneum*. **A** The differential expression profiles of *SsYABBYs* in stem from juvenile to mature. **B** The expression levels of *SsYABBYs* were analyzed in different leaf segments. **C** Expression pattern of *SsYABBY* genes during the different stages of ovule development. **D** Expression patterns of *SsYABBY* genes were performed in root, stem, and leaf by RT-qPCR analysis. **E** The differential expression levels of *SsYABBYs* in different ovules stages were confirmed by RT-qPCR analysis

To investigate the putative function of *SsYABBY* genes, the spatiotemporal expression patterns of all 7 *SsYABBY* genes were analyzed in different development stages and different tissues. For the vegetative growth from juvenile to adult stages, three different stem development stages of stems, including seedling stem, premature stem, and mature stem, were used to analyze the expression levels of all *SsYABBY* genes (Fig. 5A; Table S4). *YAB2* clade members *SsYABBY3*, *SsYABBY4*, and *SsYABBY5* expressed highly in the seedling stem. Among the *SsYABBYs*, the expression level of *SsYABBY4* was highest during the stem development progress, while *SsYABBY1*, *SsYABBY2*, *SsYABBY6*, and *SsYABBY7* expressed lower, suggesting their limited roles in stem development stages.

To better reveal the function of *SsYABBY* genes during the photosynthesis, the expression level of *SsYABBYs* was checked in leaf segments with a continuous leaf developmental gradient (basal zone, transitional zone, maturing zone, and mature zone) (Fig. 5B; Table S5). The results showed that *SsYABBY3*, *SsYABBY4*, and *SsYABBY5* were mainly expressed from the basal zone to the mature zone. *SsYABBY3* and *SsYABBY4* showed higher expression levels in the basal zone, and the expression levels decreased gradually as the leaf matured. *SsYABBY5* showed an increased expression from the basal zone to the transitional zone and decreased expression from the transitional zone to the mature zone. The expression levels of *SsYABBY1*, *SsYABBY2*, *SsYABBY6*, and *SsYABBY7* were low or undetectable in these leaf segments, except for *SsYABBY7*, which was expressed only in the basal zone, suggesting their functional limitation during the photosynthesis.

For the meristematic and reproductive tissues, the functional divergence of *SsYABBY* genes was analyzed in sugarcane ovaries at 5 different ovule development stages (AC, MMC, Meiosis, Mitosis, and Mature). *SsYABBY2* and *SsYABBY5* were mainly expressed in these different ovule development stages. The expression level of *SsYABBY1* was the highest in the MMC stage but lowest in the Mature stage. Notably, *SsYABBY3*, *SsYABBY4*, *SsYABBY6*, and *SsYABBY7* were enriched in the mitosis stage but were lower or undetectable in AC and MMC stages of ovule development (Fig. 5C, E; Table S6).

For the root, stem, and leaf tissues, the expression levels of *SsYABBY* genes were investigated by RT-qPCR analysis with primers in Table S7. As shown in Fig. 5D, all 7 *SsYABBYs* except *SsYABBY7* were predominately expressed in leaf tissues. *SsYABBY1*, *SsYABBY2*, *SsYABBY6*, and *SsYABBY7* were mainly expressed in root, and *SsYABBY2*, *SsYABBY4*, *SsYABBY6*, and *SsYABBY7* were expressed in stems. All together, *SsYABBY* genes were lowly expressed in roots, and *SsYABBY3*, *SsYABBY4*, and *SsYABBY5* were mainly responsible for the stem and leaf development. *SsYABBY2*, *SsYABBY5*, and *SsYABBY7* were mainly associated with ovule development. (Fig. 5D).

### *SsYABBY2* regulates asymmetric leaf division and ovule polarity establishment

**Fig. 6:**
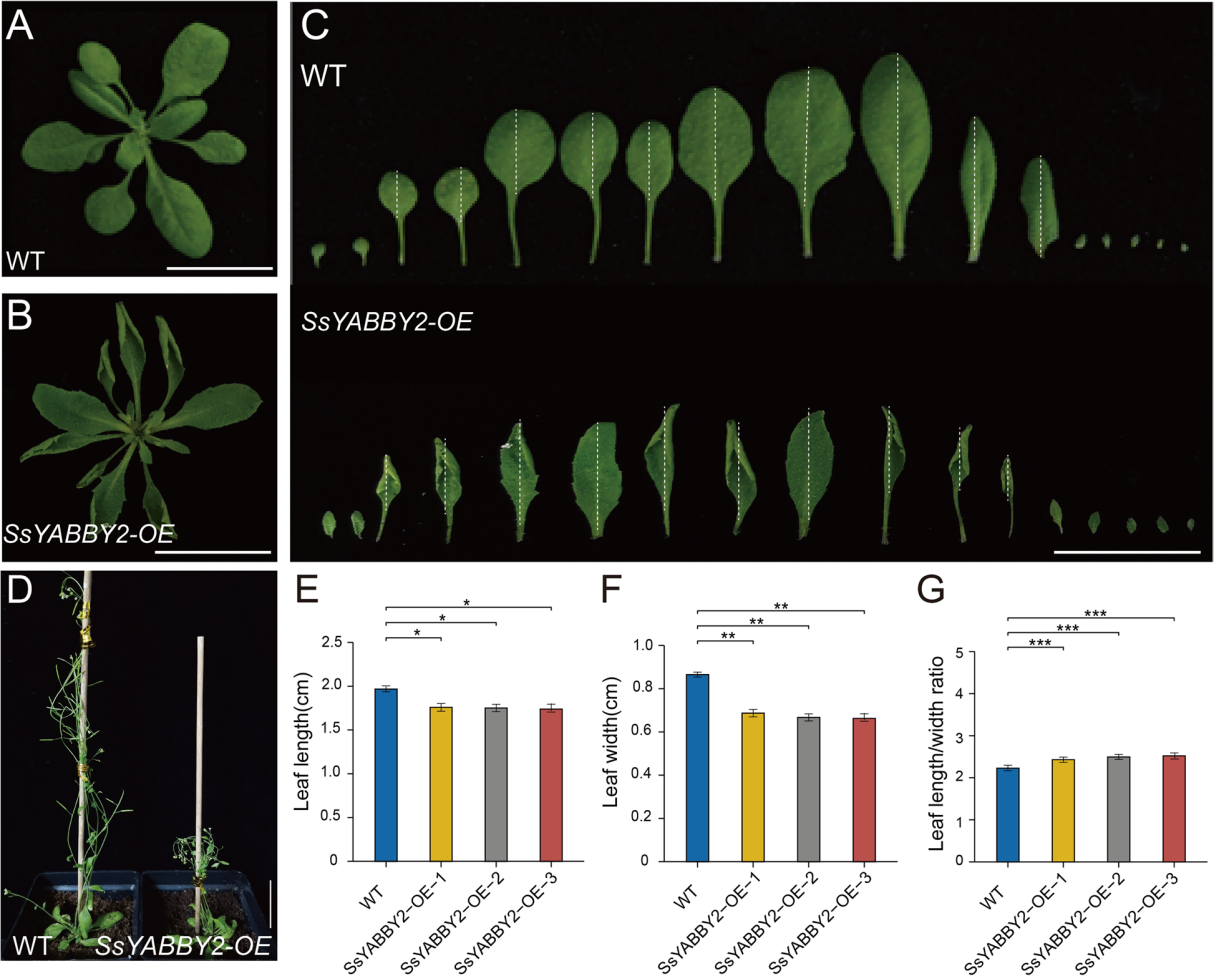
Phenotypic analysis of *SsYABBY2* transgenic plants. **A**-**C** The leaves of *SsYABBY2* transgenic plants (three-week-old) showed curled leaves compared with WT. **D** The *SsYABBY2* transgenic plants showed retarded growth. **E**-**G** Leaf length and leaf width as well as their ratio showed leaf curling in *SsYABBY2* transgenic plants. The leaf length measurement was indicated by the white dotted line. WT: wild-type, *SsYABBY2-OE*: overexpression of *SsYABBY2* in *Arabidopsis*. A Paired-samples t-test was selected for statistical analysis. * represents *p*< 0.05, ** represents *p*<0.01, *** represents *p*<0.001

The expression patterns of *SsYABBY* genes suggested that *SsYABBY* are responsible for the development of the vegetative and reproductive tissues. To further study the functional roles of *SsYABBYs* in the vegetative and reproductive tissues, *SsYABBY2*, which belongs to the *CRC* clade, was selected to explore its role in leaf development and ovule development using cross-species expression and/or complementation methods. The full-length *SsYABBY2* cDNAs were introduced into *Arabidopsis* wild-type (WT) and *crc* mutant plants under the control of the constitutive 35S promoter using the floral dip method. A total of 10 WT-overexpression and 12 *crc* mutant complementary T3 transgenic lines were obtained, and three corresponding independent lines were used for further phenotype investigation. Compared with WT plants, the *SsYABBY2-OE* transgenic plants (3-week-old) showed prominent inward curled rosette leaves (Fig. 6A, B), and the leaves were curled from the abaxial side to adaxial side and became slender configuration (Fig. 6B, C). Moreover, the leaf abaxial-adaxial polarity deficiency phenotype became more severe with the leaf development from juvenile into mature (Fig. 6C). Compared with WT, the leaf length and leaf width of overexpression lines was significantly decreased (Fig. 6E-F), however, the leaf length/width ratio was slightly increased (Fig. 6G). Additionally, the overexpression plants (6-week-old) exhibited growth retardation, delayed flowering time, and slightly reduced fertility (Fig. 6D).

**Fig. 7:**
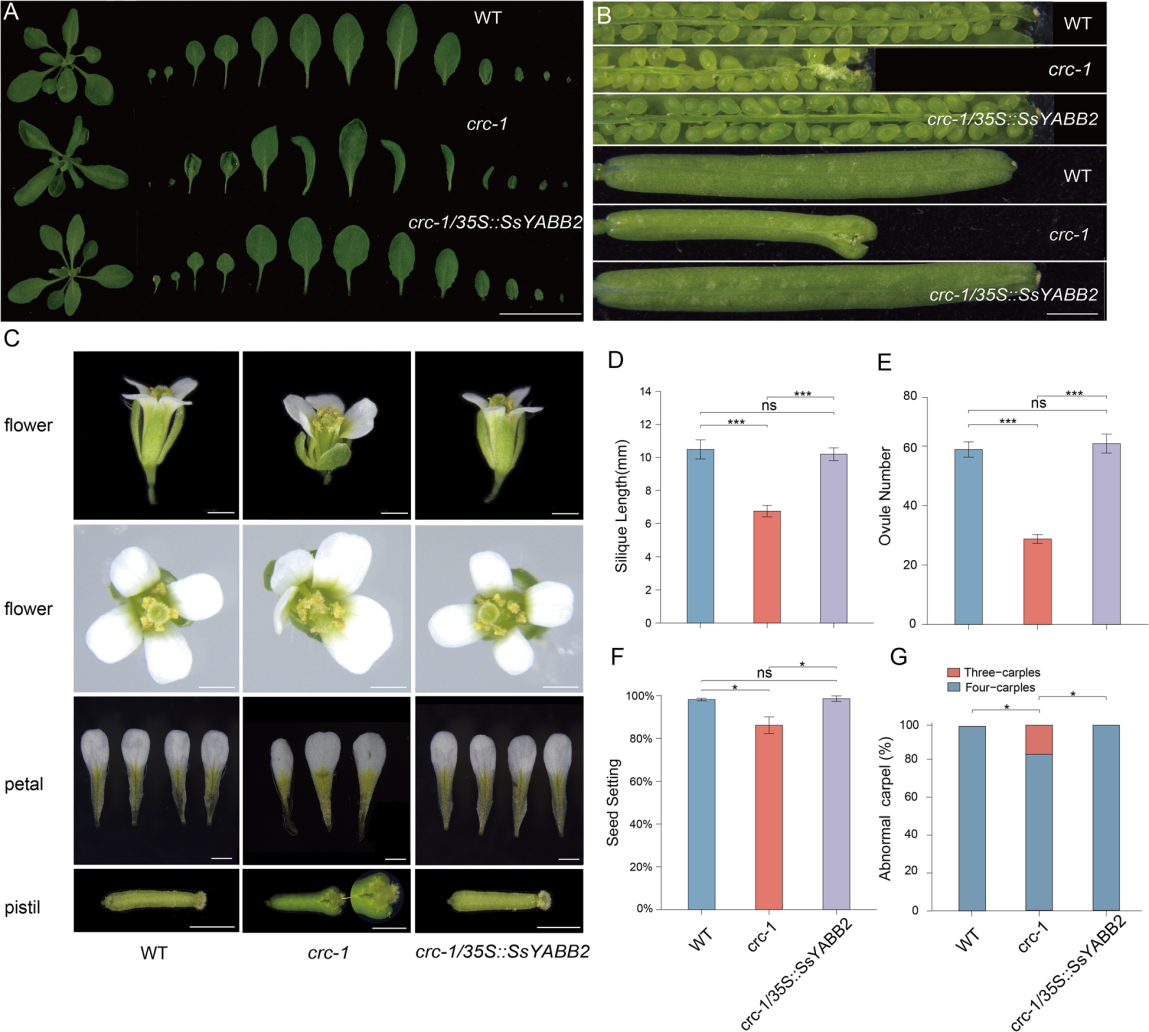
*SsYABBY2* can completely rescue *crc*-1 mutant. **A**, **B** The phenotype of rosette leaves and siliques of 14-day-old seedlings in WT, *crc*-1, and *35S::SsYABBY2*-GFP/*crc*-1. **C** The phenotype of flower, petal, and pistil was comparably analyzed in 21-day-old plants in WT, *crc*-1, and *35S::SsYABBY2*-GFP/*crc*-1. **D**-**G** Comparable analysis of silique length, ovules number, seed set, and abnormal carpel in WT, *crc*-1, and *35S::SsYABBY2*-GFP/*crc*-1 plants. WT: wild-type, *SsYABBY2-OE*: overexpression of *SsYABBY2* in *Arabidopsis crc* mutant. Paired samples t-test was selected for statistical analysis. * represents *p*< 0.05, ** represents *p*<0.01, *** represents *p*<0.001

In *Arabidopsis, CRC* plays an essential role in carpel morphogenesis and nectary specification [11]. Loss of *CRC* function resulted in a series of aberrant phenotypes, including cotyledons curled, nectaries loss, reduced ovule number, medially split, and reduced style tissue. We introduced the full-length of *SsYABBY2* cDNAs driven by the constitutive 35S promoter into *Arabidopsis crc* mutant plants and obtained 12 *crc* mutant complemental lines in the T3 generation. As shown in Fig. 7, the defective phenotypes of *crc* mutant were completely recovered by expression of *35S::SsYABBY2*. For example, the compact inflorescence, petal number, and style cracking were recovered in *35S::SsYABBY2* complemental lines (Fig. 7). These results show functional conservation of *SsYABBY2* in the establishment of leaf asymmetric division and carpels polarity.

### SsYABBYs interaction protein prediction

**Fig. 8:**
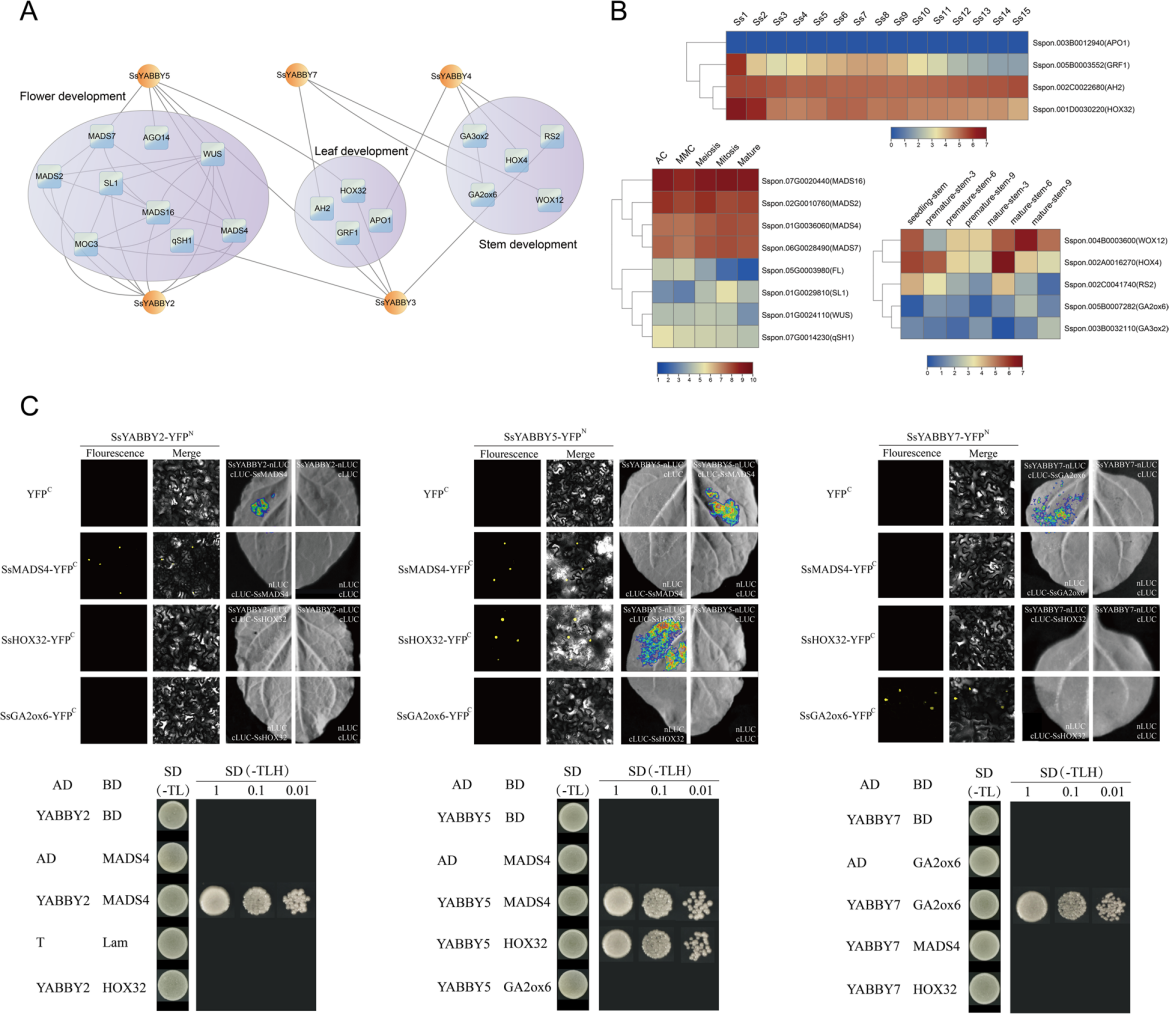
The interaction map of *YABBY* genes in *S. spontaneum*. **A** The interaction proteins of SsYABBYs were predicted by PPI analysis. The correlation network of *SsYABBY* genes was constructed with a protein-protein interactions algorithm in Cytoscape 3.8.2, each node represents a gene and the connecting lines between genes represent coexpression correlations. **B** Expression patterns of candidate interactors of SsYABBY1-7 proteins in flower development, leaf development and stem development. **C** The protein interaction between these candidates and SsYABBY proteins was confirmed by the BiFC, Y2H, and LUC assays. The results showed that SsYABBY2 could physically interact with SsMADS4, SsYABBY5 could physically interact with SsMADS4 and SsHOX32, and SsYABBY7 could physically interact with SsGAox6

To further test the functional conservation of *SsYABBYs*, candidate interaction proteins of SsYABBYs were predicted by protein-protein interaction (PPI) analysis (Fig. 8A). As shown in Fig. 8A, *SsYABBY2* and *SsYABBY5* were highly expressed genes in the ovule and associated with several MADS-box proteins (MADS2, MADS4, MADS7, MADS16, MONOCULM3 (MOC3), STAMENLESS1 (SL1), ARGONAUTE14 (AGO14), and WUSCHEL (WUS)) to form a highly interactive cluster. These genes were connected with predominant expression in the floral organs and reproductive organ tissues, suggesting the conserved functions of *SsYABBY2* and *SsYABBY5* in the development of reproductive tissues (Fig. 8A, Table S8-S10). In leaf for the adaxial-abaxial polarity development, 4 proteins (AH2, HOX32, GRF1, and APO1) were identified as the candidate interactors of SsYABBY3/4/5, with different levels of connectivity among each other (Fig. 8A, Table S8-S10). For stem development, hormone metabolism-associated proteins, such as GA2ox6, GA3ox2, HOX4, WOX12, and RS2, were among the candidate interactions of SsYABBY3/4 proteins (Fig. 8A, Table S8-S10). Interestingly, the expression levels of all the interacting proteins were also enriched in these corresponding tissues (Fig. 8B, Table S8-S10). Moreover, Yeast-2-hybrid (Y2H), bimolecular fluorescence complementation (BiFC), and Dual-luciferase reporter assays (LUC) assays were adopted to confirm these protein interactions. As expected, SsYABBY2 directly interacted with SsMADS4, SsYABBY5 physically interacted with SsMADS4 and SsHOX32, and SsYABBY7 can interact with SsGAox6 (Fig 8C).

**Fig. 9:**
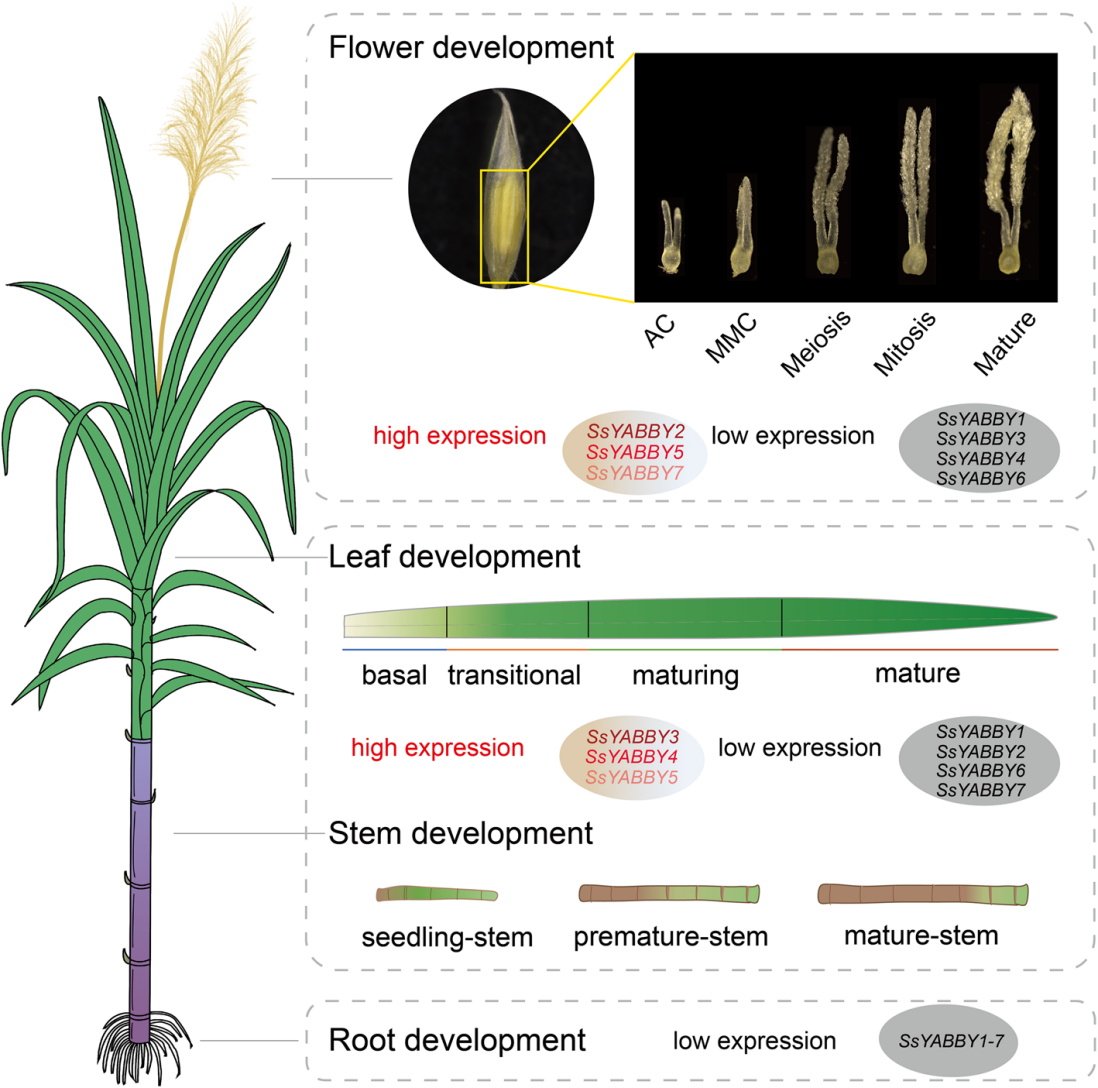
The potential functions of *YABBY* genes in *S. spontaneum*. Schematic models for expression patterns and functions analysis of *SsYABBY* genes during vegetative and reproductive tissues. The sugar transporter and lateral organs polarity establishment were analyzed based on gene expression profiles and functional complement analysis across the stems and leave development and mutant defects recovery in *S. spontaneum*. The *SsYABBYs* marked with red color were highly expressed genes, the genes marked with black color mean lower expressed genes

## Discussion

During the development, cells acquiring distinct fates greatly depends on the cell polarity establishment and maintenance [1, 3]. In plants, cell polarity is a fundamental feature in almost all aspects of cellular function, including cell expansion, division, differentiation, and morphogenesis [2, 3, 29]. In many species, the YABBY transcription factors were reported to play fundamental roles in the adaxial-abaxial polarity establishment and lateral organs development [2, 10–12, 15–17]. In this study, a total of 20 *YABBY* genes, including alleles, were genome-widely identified in *S. spontaneum*. More *SsYABBYs* existence in the sugarcane genome compared with the number of *YABBY* genes in *S. bicolor* (8) and *O. sativa* (8) indicated that the *SsYABBY* genes underwent the gene duplication events along with the sugarcane genomic autopolyploidization.

According to the phylogenetic and gene structure analysis, *SsYABBY* genes could be classified into four subgroups. Members clustered together into a subgroup shared similar gene structure and functions, indicating the functional conservation of these *SsYABBY* genes (Figs. 1 and 2). Notably, no *SsYABBY* gene could be clustered into the *YAB5* subgroup; besides, no monocotyledon *YABBY* members were found in this subgroup (Fig. 2). This result is in line with the previous reports, suggesting that the *YAB5* clade genes may generate along with the evolutionary divergence of monocotyledon and dicotyledons [8, 10, 30, 31]. The diversity of gene structures also plays an important role in expanding gene family members and the generation of novel genes. In our study, the gene structure of the *SsYABBY1-2*, *SsYABBY3-2*, *SsYABBY3-3*, and *SsYABBY3-4* was variable (Fig. 1), implying the distinct function of *SsYABBY* genes. This phenomenon may be illustrated by the gene rearrangement and/or different chromosome fragments fusion during the sugarcane genomic autopolyploidization [32, 33].

Gene duplication events are the contributors to evolutionary momentum, and duplicate genes are mainly derived from whole-genome duplication (WGD), tandem, and segmental duplication [34–36]. In this study, just one pair of tandem repeat genes was identified, while twenty-three pairs of segmental duplications were found. Furthermore, the synteny analysis between sugarcane and its relative species (rice and sorghum) displayed the close evolutional and functional relevance in three species, suggesting those *YABBY* genes shared a similar function. For example, *OsYABBY1* regulated the differentiation of reproductive cells [37]. *OsDL* controls the stamen and carpel specification as a novel gene in rice [20]. Interestingly, a couple of *SsYABBYs* showed collinear regions with *OsYABBYs*, suggesting the roles of *SsYABBY* genes in cell differentiation and carpel polarity.

To further understand the functional divergence of *SsYABBY* genes, transcriptome profiles of sugarcane stems from juvenile to mature, leaves from basal zone to apex zone, and different ovule development stages were investigated to clarify their roles of *SsYABBY* genes in sugar transport, photosynthesis, and establishment of leaf and ovule polarity. The expression levels of *SsYABBY* genes showed that *YAB2* clade members (*SsYABBY3*, *SsYABBY4*, and *SsYABBY5*) were highly expressed in the seedling-stem stage (Fig. 5A). Similarly, genes belonging to this clade showed high expression in four zones of leaf segments, and enriched in the basal zone and transition zone (Fig. 5B). These results implied the potential function of these genes in sugar transport and photosynthesis. Previous functional study of *YABBY* genes in *Incarvillea arguta* showed that overexpression of *IaYAABY2* altered the leaf and sepal polarity and increased the anthocyanin content level and photosynthesis capability of plants [38]. Additionally, *YAB2*, *YAB3*, and *FIL* expressed in the abaxial domain of lateral organs, including cotyledons, leaves, and floral organs in *Arabidopsis*; thus, they worked as “vegetative *YABBY* genes” [14]. However, for reproductive tissues, *SsYABBY2* and *SsYABBY5* (belonging to *CRC* and *YAB2* clade) were predominately expressed during ovule development. *SsYABBY3* and *SsYABBY7* expressed weakly from AC to mature stages (Fig. 5C, E). The differential expression levels of *SsYABBY* genes suggest that *SsYABBY* genes are potentially involved in sugar transport, leaf morphogenesis, and ovule polarity.

To better understand the functional roles of *SsYABBY* genes in the adaxial-abaxial polarity establishment and lateral organs development, the *CRC* clade gene, *SsYABBY2* was selected for further exploration of its function. Ectopic expression of *VpYABBY1* altered leaf adaxial-abaxial polarity in *Arabidopsis* [39]. Overexpression of soybean (Glycine max) *GmFILa* in *Arabidopsis* resulted in the abaxial polarity change of leaf epidermal, prolonged flowering, and inhibited apical meristem development [40]. Consistent with the previous reports, the *SsYABBY2*-OE lines showed the prominent curled rosette leaves from the abaxial side to the adaxial side, and finally, leaves grow into a slender configuration (Fig. 6A-C). *SsYABBY2*-OE lines also showed meristem inhibition and delayed flowering (Fig. 6D).

In addition, *CRC* clade is specifically expressed in reproductive organs, such as carpels and ovules, so-called “flower specific YABBY genes” in *Arabidopsis* [41, 42]. In pea, the ortholog of *CRC* is also involved in carpel morphogenesis [18, 19]. For monocots, such as, *Drooping Leaf* (*DL*) in rice is orthologous with *CRC*. The loss-of-function *dl* mutation caused a complete homeotic transformation of carpels into stamens [17]. In maize, the *CRC* homolog gene *DRL1* (*Drooping Leaf1*), expressed in incipient and emergent leaf primordia, modulating leaf development and plant architecture [20–22]. In our study, *SsYABBY2* was also preferentially transcribed in ovaries during ovules development (Fig. 5C), and ectopic expression of *SsYABBY2* in *Arabidopsis crc* mutant could rescue the defective phenotype of carpel dehiscent in *crc* mutant (Fig. 7B). The adaxial to abaxial curled leaves and shortened silique defects of *crc* mutant were also completely recovered by *SsYABBY2* expression (Fig. 7). Altogether these results further confirmed the functional specificity of *CRC* clade genes in the establishment and maintenance of the ovule polarity.

PPI of SsYABBYs also demonstrated the conserved function of *SsYABBY* genes for vegetative and reproductive development. For example, the MADS-box transcription factor genes *MADS6*, *MADS16*, and *MADS3* function redundantly in the identity of the carpel/ovule development and floral meristem determinacy with the *YABBY* homologous gene *DL* [43]. *OsSL1* also regulated *SPW1/OsMADS16* expression, specifying lodicule and stamen identities [44]. We also found the strong expression levels of these homologous genes in the reproductive tissues of *S. spontaneum* (Table S8-S10), validating the conserved functions of these genes in floral organs development. Previous studies reported that *AH2* deficiency leads to abaxial mesophyll cell programmed death for leaf polarity development and suppresses the abaxial development [45, 46]. *OsAPO1* controls spikelet number and overexpression of *APO1* causes an increase in inflorescence branches and spikelet [47]. For stems development, some phytohormone-related genes (*HOX4*, *GA2ox6*, and *GA3ox2*) were predicted as the interacting partners of SsYABBY3/4/5 (Fig. 8A, Table S8-S10). In rice, these genes regulated gibberellin (GA) signaling and fine-tune GA responses [48–51], causing different degrees of dwarfing and increasing the number of tillers, which suggested that the GA signal pathway may play a crucial role during the stem development of *S. spontaneum*.

## Conclusion

In conclusion, the present study identified and analyzed 20 *SsYABBY* genes in the *S. spontaneum* genome, which were classified into 5 subgroups. Phylogenetic and syntenic analysis verified that gene duplication contributed to expanding the *SsYABBY* gene family. Expression pattern analysis suggested that *SsYABBY3/4/5* plays an important role in photosynthesis. *SsYABBY2/5/7* may be responsible for leaf adaxial–abaxial polarity and carpel polarity establishment. Functional characterization indicated that *SsYABBY2* is involved in the leaf morphogenesis and carpel polarity establishment (Fig. 9). Taken together, this systematic study provides a fine-scale map of transcriptional changes of *SsYABBY* genes in global tissues in sugarcane and uncovers a large number of candidate developmental regulators orchestrating the development of the different tissues in *Saccharum spp*.

## Material and methods

### Plant materials

The sugarcane (*S. spontaneum* L.) cultivar Yuetang 91-976 was grown and collected by State Key Laboratory for Conservation and Utilization of Subtropical Agro-Bioresources (Guangxi, China), and samples from this cultivar were used for all experiments. As previously reported [52], samples were collected from 5 different development stages of the ovule (including Archesporial Cell (AC), Megaspore Mother Cell (MMC), Meiosis, Mitosis, and Mature and 4 different leaf developmental stages (basal zone, transitional zone, maturing zone, mature zone), as detailed described by Mao et al., (2021) and Zhang et al., (2016). In this present study, *Arabidopsis thaliana* plants (Col-0) and *crc-1* mutant ordered from AraShare, were grown under 16 h light/8 h dark photoperiod conditions at 22°C.

### Sequence identification of *YABBY* genes in *S. spontaneum*

The Saccharum Genome database (http://sugarcane.zhangjisenlab.cn/sgd/html/index.html) was used for retrieving the genomic sequences [32]. The sequence data of *Sorghum bicolor* and other species were downloaded from Phytozome v13 [53]. The HMM model of the YABBY domain (PF04690) was used as the query sequence to search the sugarcane genome database. All candidate *YABBY* genes were further analyzed by the CDD program to confirm the C2C2 domain and YABBY domain. The SsYABBY proteins information such as isoelectric point (pI), molecular weight (MW), and protein length were predicted using ExPASy-Compute pI/MW.

### Sequence alignment, gene structure, and phylogenetic analysis of *SsYABBY* genes

Multiple sequences alignment of YABBY protein from *A. thaliana*, *O. sativa*, *V. vinifera*, *S. bicolor*, and *S. spontaneum* were calculated by MUSCLE and visualized by Jalview [54]. The default setting parameters was the maximum number (20), minimum width (6), and maximum width (50). The structure of Ss*YABBYs* was displayed using the TBtools software [55]. The *cis*-acting elements of YABBY gene promoters were predicted by PlantCARE, and transcription factors were predicted by PlantRegMap [56]. The selection and substitution rates, the non-synonymous (*Ka*), synonymous (*Ks*), and *Ka*/*Ks* substitution ratios of the homologous gene pairs of sugarcane and sorghum were calculated by Ka/Ks calculation program. A phylogenetic tree was constructed by the MEGA 7.0 program using the ML method based on the JTT substitution model [54].

### Collinearity analysis of *SsYABBY* genes

The loci of *SsYABBY* genes were retrieved from the sugarcane annotation GFF3 files. TBtools were used to visualize gene locations on the sugarcane chromosomes [55]. For collinearity analysis, the gene pairs with a cut-off e-value of 1×10^−5^, used for MCScanX analysis, generating collinearity blocks. CIRCOS software was used for collinearity mapping within the sugarcane, rice, and sorghum genome [57].

### Transcriptome profiles analysis of YABBYs and RT-qPCR

The RNA-seq data of leaf development (including basal zone, a transitional zone, a maturing zone, a mature zone) were downloaded from the Saccharum Genome database (http://sugarcane.zhangjisenlab.cn/sgd/html/index.html). The RNA-seq data of female reproductive development (including AC, MMC, Meiosis, Mitosis, and Mature) were downloaded from the European Nucleotide Archive (ENA, accession number PRJEB44944). The RNA-seq clean reads were obtained by Trimmomatic software and mapped to the reference genome by Hisat2 [58]. DESeq2 and fragments per kilobase million values (FPKM) were used to analyze gene expression levels [59]. The FPKM value of *YABBY* genes was transformed using the log2-transformed method, and the expression patterns were generated using the heatmap package in R software.

RT-qPCR assays were performed in three different tissues (root, stem, leaf) and five female reproductive stages (AC, MMC, Meiosis, Mitosis, Mature). The total RNA was isolated using RNA Extraction Kit (R6827-01, OMEGA, China), and further analyzed by gel electrophoresis and NanoDrop2000 (Thermo Fisher, China). First-strand cDNA was synthesized with the TransScript All-in-One First-Strand cDNA Synthesis SuperMix for qPCR (TransGen Biotech). RT-qPCR was carried out using SYBR-green fluorescence (TaKaRa Biotechnology) on a Multicolor Real-Time PCR Detection System (Bio-Rad) with a 40 cycle of 95°C for 30 s; 95°C for 5 s and 60°C for 40 s. Each sample was replicated three times, and the 2^-ΔΔCT^ method was used for calculating the gene expression levels [60].

### Vector construction, subcellular localization, and transgenic analysis

The full-length coding region of *SsYABBY* genes without terminator code was amplified using primers listed in Supplementary Table S1. The PCR fragments were cloned into the pENTR/D-TOPO vector and sequenced, and then recombined into the destination vector pGWB605 by LR reaction. The resulting plasmid pGWB605-SsYABBY-GFP and empty vector pGWB605-GFP were transformed into *Agrobacterium tumefaciens* strain GV3101 and injected into leaves of *Nicotiana benthamiana* (4-week-old). After 36-48 h treatment, GFP signals were checked under a Leica confocal microscope with excited at 514 nm. The *Agrobacterium tumefaciens* strain GV3101 with *SsYABBY2* was used to transform the *crc* mutant and wild-type plants using a floral dip procedure (Clough and Bent, 1998).

### PPI network construction of SsYABBYs

A precomputed global resource, the Search Tool of the Retrieval of Interacting Genes (STRING) (http://string-db.org/) database is used for evaluating protein-protein interaction (PPI) information [61]. We used the STRING online tool to predict the PPI pairs of SsYABBY proteins with a combined score of > 0.4. Cytoscape v3.8.2 was used for PPI network construction (https://cytoscape.org/) [62].

### Yeast two-hybrid assay

The full-length CDS of *SsYABBY2*, *SsYABBY5*, and *SsYABBY7* was cloned into the pGADT7 vector at the *Nde*I site for fusion with the GAL4 activation domain. The full-length CDS of *SsMADS4*, *SsHOX32*, and *SsGAox6* were cloned into the pGBKT7 vector at the *Nde*I site for fusion with the GAL4 DNA-binding domain. Approximately 0.1 μg plasmids of bait and prey were co-transformed into the yeast strain AH109 using the Matchmaker™GAL4 Two-Hybrid System according to the manufacturer’s instructions (Clontech, USA). After growth at 28 °C for 3 days, yeast transformants were diluted and transferred to medium supplemented with SD/-Leu-Trp for growth. The yeast transformants on medium supplemented with SD/-Leu-Trp-His-Ade and 3-amino-1,2,4-triazole (3-AT) for protein interaction selection. The primers used to generate the constructs are listed in Table S7.

### Dual-luciferase reporter assay

The full-length CDS of *SsYABBY2/5/7* without the stop codon was cloned into the pCAMBIA 1300-nLUC vector to generate the pCAMBIA 1300-*SsYABBY2/5/7*-nLUC construct. The full-length CDS of *SsMADS4*, *SsHOX32*, and *SsGAox6* without the stop codon was cloned into the pCAMBIA 1300-cLUC vector to generate the related construct. Transient expression assays were performed as described previously [63]. Briefly, the recombinant constructs were transformed into *Agrobacterium* strain GV3101 and infiltrated into tobacco leaves. After 2 days of incubation, LUC and REN activities were measured using a SpectraMax® i3xMulti-Mode detection platform (Molecular Devices, USA) with a Dual-Luciferase Reporter Assay Kit (Pro-mega, USA). The LUC to REN ratio was calculated as a measure of the transcriptional activity. The primers are listed in Table S7.

### Bimolecular fluorescence complementation assay

The open reading frames of full-length *SsYABBY2/5/7*, *SsMADS4*, *SsHOX32*, and *SsGAox6* were amplified using sugarcane genomic DNA as a template. The primers are listed in Table S7. The BiFC assay was performed as previously described [64].

## Supplementary Information

**Additional file 1.**

## Data Availability

All data generated or analyzed during this study are included in this published article and its supplementary information files. The RNA-seq data of female reproductive development have been deposited in the EMBL Nucleotide Sequence Database (ENA) with accession no. PRJEB44944 (https://www.ebi.ac.uk/ena/browser/view/PRJE44944), which will be available publicly upon acceptance of the article. The RNA-seq data of leaf development were downloaded from the Saccharum Genome database (http://sugarcane.zhangjisenlab.cn/sgd/html/index.html).
