# Supplementary material for: Expression characterization and cross-species complementation uncover the functional conservation of YABBY genes for leaf abaxial polarity and carpel polarity establishment in Saccharum spontaneum
Source: BMC Plant Biol. 2022 Mar 17;22:124. doi: 10.1186/s12870-022-03501-3 (PMC8932074; doi:10.1186/s12870-022-03501-3)
Supplement: Supplementary file 1 — Additional file 1. [file 12870_2022_3501_MOESM1_ESM.pdf]

Table S1. 20 synteny gene pairs and their ka/ks rate identified by MCScanX software.

**gene pair**		**ka**	**ks**	**ka/ks**
SsYABBY2-1	*SsYABBY2-2*	0.089411	0.117058	0.763815
SsYABBY2-1	*SsYABBY2-3*	0.020584	0.046812	0.439728
SsYABBY3-1	*SsYABBY3-4*	0.055714	0.072528	0.768163
SsYABBY2-2	*SsYABBY2-3*	0.027047	0.033191	0.814894
SsYABBY3-3	*SsYABBY3-4*	0.006361	0.019718	0.322617
SsYABBY3-3	*SsYABBY5-1*	0.089411	0.117058	0.763815
SsYABBY3-4	*SsYABBY5-1*	0.055714	0.072528	0.768163
SsYABBY3-4	*SsYABBY5-2*	0.027047	0.033191	0.814894
SsYABBY5-1	*SsYABBY5-2*	0.020584	0.046812	0.439728
SsYABBY4-1	*SsYABBY4-2*	0.011719	0.022356	0.524191
SsYABBY4-1	*SsYABBY4-3*	0.069142	0.192955	0.358334
SsYABBY4-2	*SsYABBY4-3*	0.003425	0.005236	0.654108
SsYABBY4-2	*SsYABBY7-1*	0.358846	0.378846	0.947208
SsYABBY4-2	*SsYABBY7-2*	0.358846	0.527790	0.679902
SsYABBY4-2	*SsYABBY7-4*	0.450562	0.557004	0.808902
SsYABBY4-2	*SsYABBY7-5*	0.460453	0.486939	0.945608
SsYABBY7-2	*SsYABBY7-7*	0.130329	0.310200	0.420143
SsYABBY7-4	*SsYABBY7-7*	0.003096	0.010135	0.305465
SsYABBY7-5	*SsYABBY7-7*	0.135992	0.400939	0.339183
SsYABBY7-6	*SsYABBY7-7*	0.138110	0.423121	0.326407

Table S2. 19 syntenic orthologous gene pairs and their Ka/ks rate between *S. spontaneum* and *O. sativa*

Gene1	Gene2	Ka/Ks
SsYABBY2-1	*OsDL*	0.168474
SsYABBY3-2	*OsYABBY6*	0.284268
SsYABBY1-2	*OsYABBY3*	0.436073
SsYABBY2-3	*OsDL*	0.170041
SsYABBY5-1	*OsYABBY6*	0.207038
SsYABBY5-2	*OsYABBY6*	0.187680
SsYABBY4-2	*OsYABBY1*	0.134775
SsYABBY4-3	*OsYABBY1*	0.135845
SsYABBY7-1	*OsYABBY4*	0.213699
SsYABBY7-2	*OsYABBY4*	0.229579
SsYABBY7-4	*OsYABBY4*	0.283025
SsYABBY7-5	*OsYABBY4*	0.303745
SsYABBY7-6	*OsYABBY4*	0.291119
SsYABBY7-1	*OsYABBY5*	0.318572
SsYABBY7-2	*OsYABBY3*	0.073440
SsYABBY7-4	*OsYABBY5*	0.295790
SsYABBY7-5	*OsYABBY5*	0.086923
SsYABBY7-6	*OsYABBY5*	0.151676
SsYABBY7-2	*OsYABBY5*	0.088685

Table S3. 12 syntenic orthologous gene pairs and their Ka/ks rate between *S. spontaneum* and *S. bicolor*

Gene1	Gene2	Ka/Ks
SsYABBY2-1	*SbDL*	0.191124
SsYABBY1-2	*SbYAB3*	0.318572
SsYABBY2-3	*SbDL*	0.073440
SsYABBY5-1	*SbYAB6*	0.293277
SsYABBY5-2	*SbYAB6*	0.086923
SsYABBY4-2	*SbYAB1*	0.151676
SsYABBY4-3	*SbYAB1*	0.118280
SsYABBY7-1	*SbYAB4*	0.094247
SsYABBY7-2	*SbYAB4*	0.069459
SsYABBY7-4	*SbYAB5*	0.060614
SsYABBY7-5	*SbYAB5*	0.044036
SsYABBY7-6	*SbYAB5*	0.044337

Table S4. FPKM values of *YABBY* genes based on RNA-seq data in different stem developmental stages of *S. spontaneum*.

Gene	seedling-stem	premature-stem-3	premature-stem-6	premature-stem-9	mature-stem-3	mature-stem-6	mature-stem-9
SsYABBY1	1.17	0.00	0.00	0.00	0.00	0.00	0.00
SsYABBY2	1.53	0.44	0.00	0.00	0.00	0.00	0.00
SsYABBY3	16.10	0.47	0.00	0.00	0.36	0.14	0.00
SsYABBY4	32.92	0.00	0.94	0.00	0.47	0.53	0.00
SsYABBY5	14.19	0.00	2.61	0.82	5.35	3.09	0.00
SsYABBY6	0.05	0.00	0.00	0.00	0.00	0.00	0.00
SsYABBY7	2.82	0.12	0.49	0.51	0.47	0.69	0.63

Table S5. FPKM values of *YABBY* genes based on RNA-seq data in different leaf segments of *S. spontaneum*.

Gene	basal zone			trasitional zone			maturing zone					mature zone			
	Ss1	Ss2	Ss3	Ss4	Ss5	Ss6	Ss7	Ss8	Ss9	Ss10	Ss11	Ss12	Ss13	Ss14	Ss15
SsYABBY1	2.08	0.95	1.13	1.84	0.52	0.17	0.04	0.02	0.01	0.00	0.00	0.00	0.00	0.00	0.00
SsYABBY2	3.64	0.70	0.36	0.19	0.00	0.13	0.10	0.05	0.14	0.07	0.02	0.00	0.00	0.00	0.00
SsYABBY3	41.05	53.19	40.02	26.91	15.72	10.31	8.04	5.24	4.33	5.71	2.04	0.71	3.69	3.92	6.19
SsYABBY4	48.42	54.19	51.91	37.94	38.84	36.41	30.91	30.94	22.40	16.11	18.24	14.68	14.96	15.35	14.72
SsYABBY5	7.12	11.57	14.81	16.98	18.80	15.49	15.36	12.24	12.15	10.96	9.51	9.61	9.42	11.03	10.22
SsYABBY6	0.03	0.00	0.00	0.03	0.00	0.00	0.03	0.00	0.00	0.00	0.00	0.00	0.00	0.00	0.00
SsYABBY7	15.64	2.32	0.75	0.17	0.03	0.14	0.15	0.19	0.17	0.27	0.17	0.19	0.11	0.07	0.21

Table S6. FPKM values of *YABBY* genes based on RNA-seq data in different ovule stages of *S. spontaneum*.

Gene	AC	MMC	Meiosis	Mitosis	Mature
SsYABBY1	2.76	6.90	3.57	2.97	1.64
SsYABBY2	90.28	72.38	159.78	160.37	149.14
SsYABBY3	12.97	9.16	17.79	31.88	16.46
SsYABBY4	1.18	0.94	0.36	1.89	1.38
SsYABBY5	62.66	30.00	49.81	67.12	67.66
SsYABBY6	0.17	0.21	1.31	2.37	2.01
SsYABBY7	21.16	22.50	21.55	30.94	11.94

Table S7. The primers used in this study.

Gene Name	Forward/Reverse Primers	Purpose
SsYABBY1	CGGGTAAAGGGACCAAAGAG<br>CAAAGTGGATGTGTGGAAAGTG	RT-qPCR
SsYABBY2	GGAGGAGATTCAACGCATCAA<br>GCCCTATCTACCTCCAGATCAA	RT-qPCR
SsYABBY3	CACTGCAACTTCTGCAACAC<br>GTCTGGGTTGCTTGCTTTAATC	RT-qPCR
SsYABBY4	TTGTCCAGAACAGAGGGTTTC<br>TCTCTGGGTTGCTCATCTTTATC	RT-qPCR
SsYABBY5	CAGTGCAACTTCTGCAACAC<br>CCTTGATCCTGCGTATCTCTTC	RT-qPCR
SsYABBY6	GTAACAACGGCTTGTCTCCT<br>CTGGGATGTCTCGCCTTAATC	RT-qPCR
SsYABBY7	AACCTACTCTCCGTCAACCT<br>AGTGGATGTGTGGGAAATGG	RT-qPCR
SsYABBY2	catggaggccagtgaattcATGGATATGGTTTCGCAGTC<br>gtatcgatgcccacccgggtgGATGCTGCGCTCAATCTGCT	Y2H
SsYABBY5	catggaggccagtgaattcATGTCGTCGGCCCAAATCGC<br>gtatcgatgcccacccgggtgCCGAAAAAGATCCATGGTTTCTAC	Y2H
SsYABBY7	catggaggccagtgaattcATGTCGTCCTCATCCTCGTC<br>gtatcgatgcccacccgggtgGAATGGAGTGATGCCCATGT	Y2H
SsMADS4	catatggccatggaggccgaattcATGGGTCGCGGCAAGGTGGA<br>caggtcgacggatccccggTATCCATCCAGATCGACCGT	Y2H
SsHOX32	catatggccatggaggccgaattcATGGCGATGGTGGTGGTCGG<br>caggtcgacggatccccggCACGAAGGACCAGTTCACGA	Y2H
SsGAox6	catatggccatggaggccgaattcATGTGTTACGTAGCTGCCGC<br>caggtcgacggatccccggATCTTTACGGCAAAATGACTGG	Y2H
SsYABBY2	tacgcgtcccggggcggtaccATGGATATGGTTTCGCAGTC<br>atttgttggatcccgggtaccGATGCTGCGCTCAATCTGCT	LUC
SsYABBY5	tacgcgtcccggggcggtaccATGTCGTCGGCCCAAATCGC<br>atttgttggatcccgggtaccCCGAAAAAGATCCATGGTTTCTAC	LUC
SsYABBY7	tacgcgtcccggggcggtaccATGTCGTCCTCATCCTCGTC<br>atttgttggatcccgggtaccGAATGGAGTGATGCCCATGT	LUC
SsMADS4	acgggggacgagctcggtaccATGGGTCGCGGCAAGGTGGA<br>cgcgtacgagatctggtcgacTATCCATCCAGATCGACCGT	LUC
SsHOX32	acgggggacgagctcggtaccATGGCGATGGTGGTGGTCGG<br>cgcgtacgagatctggtcgacCACGAAGGACCAGTTCACGA	LUC
SsGAox6	acgggggacgagctcggtaccATGTGTTACGTAGCTGCCGC<br>cgcgtacgagatctggtcgacATCTTTACGGCAAAATGACTGG	LUC
SsYABBY2	gaacacgggggactctagaATGGATATGGTTTCGCAGTC<br>gagcccggttaactctagaGATGCTGCGCTCAATCTGCT	BiFC
SsYABBY5	gaacacgggggactctagaATGTCGTCGGCCCAAATCGC<br>gagcccggttaactctagaCCGAAAAAGATCCATGGTTTCTAC	BiFC
SsYABBY7	gaacacgggggactctagaATGTCGTCCTCATCCTCGTC<br>gagcccggttaactctagaGAATGGAGTGATGCCCATGT	BiFC
SsMADS4	gaacacgggggactctagaATGGGTCGCGGCAAGGTGGA<br>gagcccggttaactctagaTATCCATCCAGATCGACCGT	BiFC
SsHOX32	gaacacgggggactctagaATGGCGATGGTGGTGGTCGG<br>gagcccggttaactctagaCACGAAGGACCAGTTCACGA	BiFC
SsGAox6	gaacacgggggactctagaATGTGTTACGTAGCTGCCGC<br>gagcccggttaactctagaATCTTTACGGCAAAATGACTGG	BiFC

Table S8. The interaction proteins of SsYABBY2/5/7 and their FPKM value.

Gene abbreviation	Rice gene id	Sugarcane gene id	AC	MMC	Meiosis	Mitosis	Mature
MADS2	*LOC_Os01g66030.1*	*Sspon.02G0010760*	327.30	259.02	336.30	247.88	269.25
MADS4	*LOC_Os05g34940.1*	*Sspon.01G0036060*	163.10	157.46	202.73	238.52	224.51
MADS7	*LOC_Os08g41950.1*	*Sspon.06G0028490*	184.15	149.31	220.26	238.49	216.73
MADS16	*LOC_Os06g49840.1*	*Sspon.07G0020440*	510.31	370.48	503.28	565.98	506.79
WUS	*LOC_Os01g03840.1*	*Sspon.01G0024110*	18.92	19.11	22.46	20.74	8.38
MOC3	*LOC_Os04g56780.1*	*Sspon.05G0003980*	23.82	26.39	12.00	3.80	2.36
qSH1	*LOC_Os04g51000.1*	*Sspon.07G0014230*	38.91	29.80	27.85	24.91	19.96
SL1	*LOC_Os01g62920.1*	*Sspon.01G0029810*	7.92	5.96	19.55	38.06	18.11

Table S9. The interaction proteins of SsYABBY3/4/5 and their FPKM value.

Gene abbreviation	Rice gene id	Sugarcane gene id	basal zone			trasitional zone			maturing zone					mature zone			
			**Ss1**	**Ss2**	**Ss3**	**Ss4**	**Ss5**	**Ss6**	**Ss7**	**Ss8**	**Ss9**	**Ss10**	**Ss11**	**Ss12**	**Ss13**	**Ss14**	**Ss15**
GRF1	*LOC_Os02g53690.1*	*Sspon.005B0003552*	59.77	12.47	7.15	9.88	12.92	15.90	18.59	18.33	14.98	9.57	6.48	3.80	3.00	2.27	2.21
APO1	*LOC_Os06g45460.1*	*Sspon.003B0012940*	0.00	0.00	0.00	0.00	0.00	0.00	0.00	0.00	0.00	0.00	0.00	0.00	0.00	0.00	0.00
AH2	*LOC_Os09g23200.1*	*Sspon.002C0022680*	46.08	44.68	39.85	35.36	36.32	33.83	33.86	33.33	32.12	32.79	34.06	36.44	39.01	39.86	43.74
HOX32	*LOC_Os03g43930.1*	*Sspon.001D0030220*	95.44	65.01	27.99	25.17	28.37	34.73	34.02	28.25	29.72	25.71	25.43	23.80	21.30	21.62	16.56

Table S10. The interaction proteins of SsYABBY3/4/5 and their FPKM value.

Gene abbreviation	Rice gene id	Sugarcane gene id	seedling-Sstem	premature-stem-3	premature-stem-6	premature-stem-9	mature-stem3	mature-stem6	mature-stem9
GA2ox6	*LOC_Os04g44150.1*	*Sspon.005B0007282*	0.19	1.86	1.44	0.26	1.60	4.23	1.29
GA3ox2	*LOC_Os01g08220.1*	*Sspon.003B0032110*	1.40	0.91	0.51	1.91	0.04	1.12	4.41
HOX4	*LOC_Os09g29460.1*	*Sspon.004B0003600*	43.13	3.20	13.22	12.57	42.08	84.35	33.81
